# Molecular detection of antimicrobial resistance in livestock mycoplasmas: current status and future prospects

**DOI:** 10.3389/fvets.2025.1699077

**Published:** 2025-12-15

**Authors:** Kinga M. Sulyok, Zsuzsa Kreizinger, Dorottya Földi, Áron Botond Kovács, Dénes Grózner, Lucía Manso-Silván, Jade Bokma, Annet E. Heuvelink, Sara M. Klose, Anneke Feberwee, Salvatore Catania, Ana S. Ramirez Corbera, Paola K. Vaz, Cecile Boland, Kannan Ganapathy, Anne V. Gautier-Bouchardon, Claire A. M. Becker, Florence Tardy, Inna Lysnyansky, Miklós Gyuranecz

**Affiliations:** 1 Zoonotic Bacteriology and Mycoplasmology, HUN-REN Veterinary Medical Research Institute, Budapest, Hungary; 2 National Laboratory of Infectious Animal Diseases, Antimicrobial Resistance, Veterinary Public Health and Food Chain Safety, Budapest, Hungary; 3 MolliScience Kft., Biatorbágy, Hungary; 4 CIRAD UMR ASTRE, INRAE, Université de Montpellier, Montpellier, France; 5 Department of Large Animal Internal Medicine, Faculty of Veterinary Medicine, Ghent University, Merelbeke, Belgium; 6 Royal GD, Deventer, Netherlands; 7 Asia-Pacific Centre for Animal Health, Faculty of Science, University of Melbourne, Melbourne, VIC, Australia; 8 Mycoplasma Unit, WOAH Reference Laboratory for Avian Mycoplasmosis, Istituto Zooprofilattico Sperimentale delle Venezie, Buttapietra, Italy; 9 IUSA, Veterinary Faculty, University of Las Palmas de Gran Canaria, Las Palmas, Spain; 10 Sciensano, Veterinary Bacteriology, Department of Infectious Diseases in Animals, Brussels, Belgium; 11 University of Liverpool, Neston, Cheshire, United Kingdom; 12 Ploufragan-Plouzané-Niort Laboratory—Mycoplasmology, Bacteriology and Antimicrobial Resistance Unit, Anses, Ploufragan, France; 13 UMR Anses VetAgro Sup Mycoplasmoses Animales, VetAgro Sup, Université de Lyon, Marcy l’Etoile, France; 14 Mycoplasma Unit, Kimron Veterinary Institute, Beit Dagan, Israel; 15 Department of Microbiology and Infectious Diseases, University of Veterinary Medicine, Budapest, Hungary

**Keywords:** antimicrobial resistance, molecular detection, livestock mycoplasma, genome-wide association studies, SWOT

## Abstract

Pathogenic *Mycoplasma* species significantly impact livestock health, causing respiratory, articular, mammary gland, and reproductive disorders with substantial economic losses. Antimicrobials remain essential for controlling clinical signs and production losses; however, treatment efficacy is increasingly threatened by antimicrobial resistance (AMR). Phenotypic methods remain the most reliable approach for detecting AMR in *Mycoplasma* species; however, they are time-consuming, technically demanding, and results are often difficult to interpret. The absence of clinical breakpoints and limited epidemiological cut-off values (ECOFFs) further complicate AMR categorization. Advances in molecular techniques offer a promising alternative for faster AMR detection and prediction. This review summarizes current knowledge of genetic mechanisms underlying AMR in clinically important *Mycoplasma* species affecting ruminants, swine, and poultry. It highlights the role of molecular assays in identifying resistance-associated mutations. Additionally, a SWOT (Strengths, Weaknesses, Opportunities, Threats) analysis evaluates these methods’ practical applications and limitations in veterinary mycoplasmas. Finally, the potential of genome-wide association studies (GWAS) is explored as an emerging tool for linking genetic traits to phenotypic resistance patterns, offering new insights for enhancing resistance prediction in veterinary medicine.

## 1 Introduction

The *Mycoplasma* genus encompasses over 140 species, many of which are pathogenic for humans and animals posing significant health concerns in both human and veterinary medicine (1, 2). In livestock, mycoplasmas are commonly associated with respiratory, urogenital, articular and mammary gland disorders (Table 1) (3). Moreover, mycoplasmoses in livestock often occur as coinfections, either involving multiple *Mycoplasma* species or mycoplasmas in combination with other bacterial and viral pathogens. For example, in ruminants, mixed infections involving *Pasteurella multocida*, *Mannheimia haemolytica*, or bovine viral diarrhea virus can aggravate respiratory and systemic diseases (4). Similarly, in pigs, *M. hyopneumoniae* frequently acts synergistically with pathogens such as *Actinobacillus pleuropneumoniae* and porcine reproductive and respiratory syndrome virus (5), while in poultry, *M. gallisepticum* and *M. synoviae* coinfections with *Escherichia coli* or Newcastle disease virus can intensify disease severity (6). Such polymicrobial interactions complicate diagnosis, treatment, and control efforts.

**Table 1:** Clinically relevant livestock *Mycoplasma* species with published AMR-associated genetic characteristics.

Host		*Mycoplasma* species	Clinical signs or syndrome	References
Cattle		*M. bovis*	Infectious enzootic bronchopneumonia, mastitis, arthritis, otitis	(7)
		*M. mycoides* subsp*. mycoides*	Contagious bovine pleuropneumonia	(83)
		*M. bovirhinis*	Respiratory disease	(164)
		M. wenyonii*	Anemia	(149)
		Ca. M. haematobovis*		
Small ruminant		*M. agalactiae*	Contagious agalactiae	(165)
		*M. mycoides* subsp*. capri*		
		*M. capricolum* subsp*. capricolum*		
		*M. putrefaciens*		
		*M. capricolum* subsp*. capripneumoniae*	Contagious caprine pleuropneumoniae	(166)
		M. ovipneumoniae*	Atypical pneumonia	(96)
		M. ovis*	Hemolytic anemia	(150)
		Ca. M. haematovis*		
Swine		*M. hyopneumoniae*	Enzootic pneumonia	(167)
		*M. hyorhinis*	Arthritis, polyserositis	
		*M. hyosynoviae*	Arthritis, polyarthritis	
		M. suis*	Infectious anemia	(168)
Poultry	Chicken, Turkey	*M. gallisepticum*	Chronic respiratory disease, infectious sinusitis	(169)
		*M. synoviae*	Subclinical respiratory tract infections, infectious synovitis, eggshell apex abnormality syndrome in laying-hen flocks	(170)
	Chicken	*M. gallinarum*	Associated with respiratory disease	(171)
	Turkey	*M. meleagridis*	Decreased hatchability, late embryo mortality, airsacculitis, skeletal abnormalities and poor growth	(172)
		*M. iowae*		
		*M. pullorum*	Associated with embryo mortality	(173)
Waterfowl		*M. anserisalpingitidis*	Genital tract and cloaca inflammation, embryo lethality and decreased egg production	(174)

*Non-cultivable hemotropic *Mycoplasma* species (formerly *Eperythrozoon*).

Effective control of livestock mycoplasmosis relies on adequate housing and biosecurity measures as well as vaccination and appropriate antimicrobial treatments. Vaccines play a crucial role in mitigating both the clinical and economic impacts of some mycoplasma infections. Commercial attenuated live vaccines for several species have been available for many years, including *M. gallisepticum* and *M. synoviae* in poultry. However, the accessibility of certain vaccines is limited (e.g., live vaccines against the swine pathogen *M. hyorhinis* is only available in the USA, while the one against the swine pathogen *M. hyopneumoniae* is only registered in China), and in some cases (e.g., the bovine pathogen *M. bovis*) efficacy remains unproven due to the recent introduction of the vaccine and the lack of field data. As a result, disease control programs may be hindered by the limited protective efficacy of vaccines and/or their availability in endemic regions (7, 8). Therefore, antimicrobial treatment strategies are crucial during clinical outbreaks, helping to reduce the economic impact of infections. However, prolonged treatment can lead to increased costs and restrictions on the commercialization of animal products due to antimicrobial residues (9). Moreover, the use of anti-mycoplasma drugs inevitably applies selective pressure on the host’s microbiota, including zoonotic bacteria, underscoring the importance of responsible antimicrobial use to combat AMR from a *One Health* perspective (10, 11).

In addition to antimicrobial use, the transmission dynamics of *Mycoplasma* species also play a crucial role in the epidemiology of infections and the dissemination of resistance strains. These involve vertical transmission (particularly in poultry), direct horizontal spread through close contact or aerosols, introduction of carrier animals, and environmental or human-mediated dissemination. Such dynamics, varying by species and host ecology, often lead to persistent infections that can manifest as subclinical carriage or clinical outbreaks in both domestic and wild animal populations. Understanding these routes is essential to contextualize and control the spread of AMR among *Mycoplasma* populations (12, 13).

The number of antimicrobial classes expected to be active against mycoplasmas is limited, as they are naturally resistant to first-generation quinolones, antimicrobials targeting the cell wall (e.g., β-lactam antibiotics), polymixins and sulfonamides/trimethoprim (14–17). Moreover, certain species, such as *M. bovis* and *M. synoviae*, exhibit intrinsic resistance to specific drugs within the same class; for example, they are naturally resistant to erythromycin. Antimicrobials commonly used against *Mycoplasma* species typically act either on the bacterial ribosome, thereby inhibiting protein synthesis (e.g., macrolides and tetracyclines), or on DNA replication (e.g., fluoroquinolones) (14, 15). The efficacy of antimicrobials is under increased scrutiny, as rising trends of AMR—as estimated *in vitro*—have been reported globally (18) with the emergence of multidrug-resistant strains (19–21). Treatment guidelines and contraindications can further restrict veterinarians’ ability to provide treatment, even when diagnostic results are available. For instance, administering tiamulin to poultry medicated with ionophore antimicrobials is not recommended, as it may result in toxicity (22). Furthermore, the use of fluoroquinolones is prohibited in many countries for food-producing animals due to concerns over AMR and public health risks (23).

General guidelines for *in vitro* antimicrobial susceptibility testing (AST) of veterinary mycoplasmas were published in 2000 (24) and have not been revised since then. Similarly, no interpretative criteria have been established. Minimum inhibitory concentration (MIC) determination for *Mycoplasma* species is time-consuming and requires specialized laboratory skills, owing to their slow growth and fastidious nature, making routine testing impractical for immediate treatment decisions. Instead, regular AST is recommended to track resistance trends and support evidence-based treatment strategies. The MYMIC project, a consortium of 22 research institutions from 18 countries, was established to address these challenges by reviewing current veterinary and laboratory practices, compiling MIC data for key livestock pathogens, and identifying critical gaps toward establishing reference AST methods and defining ECOFFs (Jaÿ et al., accepted).^1^ Continuous surveillance across regions with varying antimicrobial usage patterns is essential for detecting shifts in resistance (25, 26) and allowing a better management of integrated systems (27).

Molecular methods can significantly reduce diagnostic turnaround time and enhance timely treatment decisions when diagnosing mycoplasma infections. A well-established example is the detection of antimicrobial susceptibility in *M. genitalium,* an emerging human pathogen which poses significant treatment challenges due to its high macrolide resistance worldwide and limited susceptibility to tetracyclines. In response to these concerns, current clinical guidelines recommend that all *M. genitalium*-positive cases undergo additional molecular testing to detect macrolide resistance-associated mutations. This genotypic approach—fully validated against gold-standard methods—enables the rapid and accurate identification of resistance, thereby supporting the timely selection of effective first-line antibiotic therapy (28). A similar approach might be valuable for livestock mycoplasmas, for example with *M. bovis*, whose many strains are resistant to the main antimicrobial families used to treat bovine respiratory diseases (29), and where alterations in genome regions are well-defined in association with macrolide and fluoroquinolone resistance, which could serve as markers for predicting resistant isolates (18).

**Figure 1:**
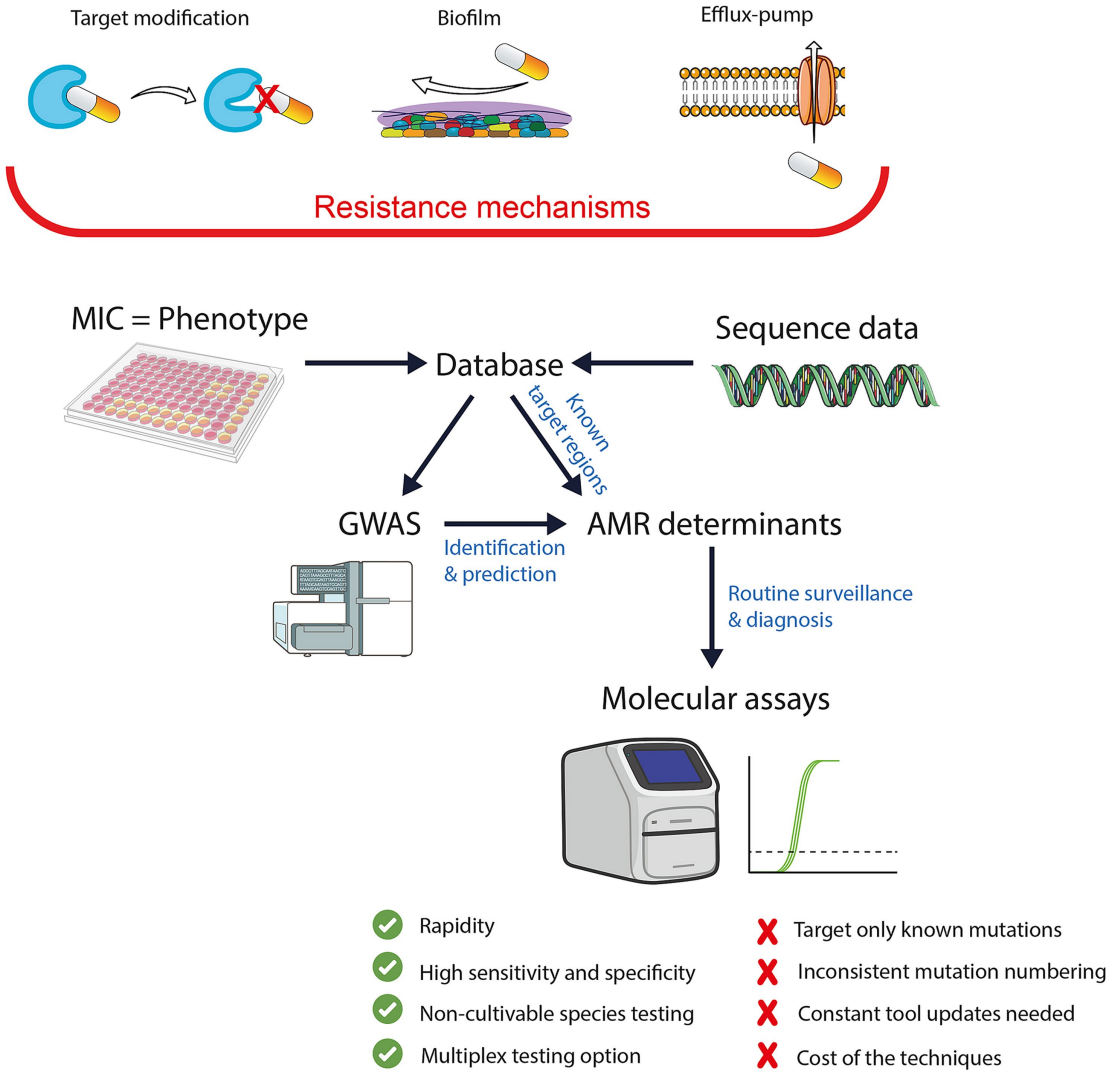
Major resistance mechanisms in livestock mycoplasmas and molecular approaches for AMR detection and GWAS-based predicting analysis. MIC, minimum inhibitory concentration, GWAS, genome-wide association study; AMR, antimicrobial resistance.

In mycoplasmas, several AMR mechanisms that are well-described in other bacteria have been also identified, including target modification, efflux pumps (30–32) and biofilm formation (33–36) (Figure 1). Among them, target modification through chromosomal mutations is the most prevalent and reported mechanism of acquired AMR in mycoplasmas to date. This review compiles the mutations reported so far in various *Mycoplasma* species, as detailed in Supplementary Tables 1–3, which cover macrolides, tetracyclines and aminoglycosides, and fluoroquinolones, respectively. The tables include information on each mutation’s occurrence by species, associated MIC values, geographic distribution, available molecular detection assays, and supporting references.

While target modification is the primary driver of high-level resistance in mycoplasmas, contributory roles of efflux, biofilm formation, and rare acquisition of resistance genes through horizontal gene transfer (HGT) events warrant consideration (Table 2). Efflux pumps contribute to AMR in livestock *Mycoplasma* species by actively exporting antibiotics, such as macrolides and tetracyclines, out of the bacterial cell. Efflux mechanisms have been identified in mycoplasmas infecting small ruminants (e.g., *Mycoplasma mycoides* subsp. *capri*), as well as in *M. hyopneumoniae* and *M. anserisalpingitidis*, showing substantial strain-to-strain variability (30–32, 37). As non-specific mechanisms of resistance, efflux pumps allow bacteria to survive at slightly elevated antimicrobial concentrations, thereby facilitating the selection of chromosomal mutations that further increase MIC levels (38). Comparative genome analyses have revealed non-synonymous single nucleotide polymorphisms (SNPs) in ABC transporter genes, which are more frequent in *M. anserisalpingitidis* strains with decreased susceptibility to several antimicrobial agents (31). Additionally, a gene expression study demonstrates up-regulation of ABC and MATE efflux pump genes in *M. hyopneumoniae* associated with reduced macrolide susceptibility (32). These findings underline the contribution of efflux pumps not only as immediate resistance mechanisms but also as facilitators for the development of higher-level resistance through genetic adaptations.

**Table 2:** Overview of non–target-modification mechanisms contributing to AMR in livestock *Mycoplasma* species.

Mechanism	Description	Findings
Efflux pump	Active export of antimicrobials via membrane transporters reduces intracellular drug concentration.	Efflux-associated decreased susceptibility to macrolides and tetracyclines reported in: *Mycoplasma mycoides* subsp. *capri* (30, 37) *M. agalactiae* (30) *M. hyopneumoniae* (32) *M. anserisalpingitidis* (31)
Biofilm formation	Surface-associated communities offering physical and metabolic protection from antimicrobials.	Correlation between *in vitro* biofilm forming ability and antimicrobial sensitivity was found in: *M. gallisepticum* (34, 36, 39) *M. synoviae* (33, 36) *M. hyopneumoniae* (35)
HGT	Acquisition of resistance-associated genes.	Conjugative chromosomal transfer: *M. agalactiae* (43) *M. bovis* (44)

Biofilm formation represents another important, though often underappreciated, contributor to antimicrobial tolerance in mycoplasmas. Biofilms are structured microbial communities encased in a self-produced polymeric matrix that attach to biotic or abiotic surfaces, enhancing resistance to environmental stresses, including antibiotics. Correlations between *in vitro* biofilm-forming ability and reduced antibiotic susceptibility have been reported for *M. gallisepticum* (34, 39), *M. synoviae* (33), and *M. hyopneumoniae* (35), whereas no such relationship was observed in *M. anserisalpingitidis* (40). Moreover, in *M. gallisepticum*, a comparative genome analysis identified several biofilm-associated genes such as *manB*, *oppA*, *oppD*, *pdh*, *eno*, *relA*, *msbA*, *deoA*, *gapA*, and *rpoS* (34). Generally, avian pathogenic *Mycoplasma* species produce more abundant biofilms than non-pathogenic isolates, and marked differences in biofilm-forming capacity have been observed both between and within species (36). Together, these findings underscore that although chromosomal mutations remain the primary mechanism of acquired resistance, non–target-modification mechanisms such as efflux and biofilm formation may play synergistic roles in shaping antimicrobial responses in *Mycoplasma* populations.

The contribution of gene acquisition to AMR in mycoplasmas is limited. For instance, target protection through the acquisition of mobile genetic elements carrying the *tet*(*M*) gene, which confers tetracycline resistance, occurs only in a few human *Mycoplasma* species (*M. hominis* and *Ureaplasma* spp.) (41, 42) and has never been reported in animal mycoplasmas. Horizontal mycoplasma chromosomal transfer of loci carrying resistance-associated mutations has been demonstrated in *M. agalactiae* laboratory model (43), and in field isolates of *M. bovis* (44), highlighting the potential for gene flow within mycoplasma populations. However, evidence for HGT of resistance determinants in swine or poultry mycoplasmas remains limited. Additionally, antibiotic inactivating enzyme genes have been identified in the prophage region of *M. bovirhinis* HAZ141 (41, 45). *M. bovirhinis* is generally not considered a primary pathogen; however, it is frequently isolated from both healthy and diseased animals, often in co-infection with other *Mycoplasma* species. The presence of prophage carrying aminoglycoside-inactivating enzyme genes in some of its strains raises the largely overlooked question of whether non-pathogenic *Mycoplasma* species may serve as reservoirs for antimicrobial resistance genes. Molecular analysis of genetic mechanisms behind AMR traits has become an essential aspect of clinical studies on bacterial infections. In situations where phenotypic testing is slow, inconclusive, or unavailable, molecular tools can identify resistance genes or mutations, directly supporting the selection of effective treatment and implementation of appropriate control measures. Moreover, molecular methods could be employed for epidemiological investigations, especially during outbreaks, to provide insights when phenotypic data is insufficient. Additionally, molecular studies contribute significantly to AMR surveillance efforts on local, national, and international levels (46). Since most resistance mechanisms in mycoplasmas are linked to point mutations, there is a need to develop and implement techniques capable of detecting specific nucleotide changes, such as restriction fragment analysis, probe-based polymerase chain reaction (PCR) assays, and other sequence-sensitive methods.

Several PCR-based molecular methods with unique capabilities are available for detecting known resistance-associated mutations in livestock mycoplasmas. These include PCR-Restriction Fragment Length Polymorphism (PCR-RFLP) (47), TaqMan SNP real-time PCR (48), Melting Curve Analysis using Hybridization Probes (49), Mismatch Amplification Mutation Assays (MAMA) (50–54), and High Resolution Melt (HRM) analysis (Supplementary Tables 4A,B) (50, 51). While these PCR-based methods can detect known resistance-associated mutations within hours rather than days, they are limited by their ability to identify only predefined mutations and require proper validation before they can be adopted as routine diagnostic tools.

GWAS serve as essential research approaches for uncovering new resistance mechanisms. By analyzing the entire genomes, GWAS identify associations between genetic variants and phenotypic resistance traits, revealing novel mutations and elucidating complex resistance pathways. GWAS expand the understanding of AMR (55–57) and guide future routine detection efforts by highlighting resistance determinants that warrant further investigation (Figure 1). As GWAS methodologies continue to evolve, they increasingly contribute to identify both known and novel resistance factors. Once key variants are identified, they can be tracked through PCR-based detection methods for routine surveillance and diagnostics. In this way, GWAS serve as a discovery platform that informs and enhances molecular detection, effectively bridging the gap between genomic research and routine laboratory application.

The present review aims to propose a comprehensive framework for the effective application of molecular assays and genetic analyses in detecting AMR in livestock mycoplasmas, with the goal of enhancing resistance surveillance and guiding antimicrobial treatment strategy. Advancing these tools is essential for mitigating the global AMR crisis and promoting sustainable livestock management.

## 2 Target modification and methods for their detection in livestock mycoplasmas

The main mechanisms of AMR in livestock mycoplasmas are based on chromosomal mutations that results in target modification. These genetic point mutations represent potential targets for molecular methods, enabling the distinction between antimicrobial wild-type and non-wild-type isolates.

The peptidyl transferase center of the 50S subunit of the ribosome is the binding site of macrolides, lincosamides, pleuromutilins and phenicols. These molecules primarily interact with the A2058 nucleotide—or neighboring nucleotides—of the *rrlA* and/or *rrlB* alleles of domain V of the 23S rRNA according to *Escherichia coli* numbering (32, 49, 51, 58–60). Additional mutations at and the G748 nucleotide—or neighboring nucleotides—in domain II of the 23S rRNA, as well as in the L4 and L22 ribosomal proteins encoded by the *rplD* and *rplV* genes were described in association with decreased susceptibility to macrolides (49, 55, 61, 62), while multiple mutations in domain V of the 23S rRNA and in the L3 ribosomal protein encoded by the *rplC* gene were linked to increased MIC values for pleuromutilins (60, 63). These shared binding sites explain the previously observed cross-resistance among these antibiotic classes (60, 63). Tetracyclines, aminocyclitols and aminoglycosides inhibit protein synthesis by binding to the A-site of the 16S rRNA within the 30S ribosomal subunit, impeding translation initiation and/or elongation (64). Several point mutations in the 16S rRNA-encoding *rrs* genes have been linked to decreased susceptibility to these antibiotic classes, with cross-resistance also reported among them (60, 65, 66).

Fluoroquinolones apply their bactericidal effect by inhibiting type II topoisomerases during bacterial replication (67). Reduced susceptibility of mycoplasmas to fluoroquinolones is mainly associated with mutations in the quinolone resistance-determining regions (QRDR) of the DNA gyrase subunits GyrA and GyrB and the topoisomerase IV subunits ParC and ParE as reviewed by Gautier-Bouchardon (18).

### 2.1 Ruminant mycoplasmas

Recent studies have shown increasing MIC values, especially for macrolides in *M. bovis* isolates from multiple countries (68–73). However, notable variability in MIC data is observed in association with geographical origin of the isolates (68–75). These findings underscore the importance of regularly monitoring antimicrobial susceptibility across various regions, where treatments practices may vary significantly. The relevant point mutations and the published detection assays targeting them are shown in Table 3 and Supplementary Tables 1–3.

**Table 3:** Existing PCR-based genotyping methods for the detection of AMR-associated mutations.

Species	Antimicrobial	Reference	Detection method	Gene	Nucleotide mutation^a^	Product	Amino acid mutation^b^
*M. bovis*	FQs	(47)	PCR-RFLP	*parC*	G250A	ParC	D84N
		(48)	TaqMan SNP PCR	*parC*	G250A	ParC	D84N
		(50)	MAMA	*gyrA*	C248T	GyrA	S83F
			HRM	*gyrA*	213–286 nt	GyrA	71–95 aa
			MAMA	*parC*	G239T	ParC	S80I
			MAMA	*parC*	G250A	ParC	D84N
	MACs	(50)	MAMA	*rrl*	G748A	23S rRNA	–
			HRM	*rrl*	672–778 nt	23S rRNA	–
		(49)	Melt curve analysis using hybridization probe	*rrl*	G748A	23S rRNA	–
	MACs+LCM	(50)	MAMA	*rrl*	A2059G	23S rRNA	–
		(49)	Melt curve analysis using hybridization probe	*rrl*	A2059G	23S rRNA	–
	50S inhibitors	(50)	HRM	*rrl*	2005–2,101 nt	23S rRNA	–
			HRM	*rrl*	2,473–2,524 nt	23S rRNA	–
			HRM	*rrl*	2,557–2,636 nt	23S rRNA	–
	TETs	(50)	MAMA	*rrs*	A965T	16S rRNA	–
			MAMA	*rrs*	A967T	16S rRNA	–
			HRM	*rrs*	993–1,005 nt	16S rRNA	–
		(49)	Melt curve analysis using hybridization probe	*rrs*	A965T	16S rRNA	–
			Melt curve analysis using hybridization probe	*rrs*	A967T	16S rRNA	–
			Melt curve analysis using hybridization probe	*rrs*	A967C	16S rRNA	–
	SPC	(50)	MAMA	*rrs*	C1192A	16S rRNA	–
		(49)	Melt curve analysis using hybridization probe	*rrs*	C1192A	16S rRNA	–
	TETs/SPC	(50)	HRM	*rrs*	1,139–1,251 nt	16S rRNA	–
*M. hyopneumoniae*	FQs	(51)	MAMA	*gyrA*	G242C	GyrA	G81A
			MAMA	*gyrA*	C247T	GyrA	A83V
			MAMA	*gyrA*	A260G	GyrA	E87G
			MAMA	*parC*	C239T/A	ParC	S80F
			MAMA	*parC*	G250A	ParC	D84N
			HRM	*parC*	239–250 nt	ParC	80–84 aa
	MACs+LCM	(51)	MAMA	*rrl*	A2059G	23S rRNA	–
*M. hyorhinis*	MACs+LCM	(53)	MAMA	*rrl*	A2058G	23S rRNA	–
*M. synoviae*	FQs	(52)	MAMA	*gyrA*	A566G^c^	GyrA	E133G
			MAMA	*gyrB*	C1247A^c^	GyrB	S401Y
			MAMA	*parC*	A253G^c^	ParC	T80I
			MAMA	*parC*	C254T^c^	ParC	T80A
			MAMA	*parE*	C260T^c^	ParE	S83F
	MACs+LCM	(52)	MAMA	*rrl*	A2059G	23S rRNA	–
	MACs	(52)	MAMA	*rplV*	A270C/T	L22	Q90H
*M. anserisalpingitidis*	MACs	(54)	MAMA	*rrl*	G748A	23S rRNA	–
			MAMA	*rplV*	A270C/T	L22	Q90H
	MACs+LCM	(54)	MAMA	*rrl*	A2058G	23S rRNA	–

PCR, polymerase chain reaction; RFLP, restriction fragment length polymorphism; SNP, single nucleotide polymorphism; MAMA, mismatch amplification mutation assay; HRM, high resolution melt; FQs, fluoroquinolones; TETs, tetracyclines; SPC, spectinomycin; MACs, macrolides; LCM, lincomycin.

^a^*E. coli* nucleotide number, ^b^*E. coli* amino acid number, ^c^ numbering according to *M. synoviae* strain MS53 (GenBank accession number: AE017245).

Reduced susceptibility to antimicrobials targeting the 50S ribosomal subunit (macrolides, lincosamides, phenicols, pleuromutilins) in *M. bovis* has been associated with point mutations mainly in the 23S rRNA genes (55–57, 60). Several alterations from positions 2058 to 2067 have been described in both clinical and *in vitro* derived laboratory-mutant strains (49, 55, 56, 60, 66, 76–80). MAMA and melting curve analysis using hybridization probes for the detection of *M. bovis* field isolates with reduced susceptibility to macrolides (associated with mutations G748A in domains II and A2059G V of the 23S rRNA, respectively) and lincosamides (A2059G in domain V of the 23S rRNA) were developed (49, 50). Association between mutations in L4 and L22 ribosomal proteins and elevated MIC of macrolides were also demonstrated in *M. bovis* (55, 80). In laboratory-derived mutant strains, mutations associated with resistance to florfenicol and pleuromutilin were detected in the 23S rRNA (60).

Decreased susceptibility to antimicrobials acting on the 30S ribosomal subunit (tetracyclines, spectinomycin, gentamicin) is associated with mutations present in genes encoding the 16S rRNA (49, 55, 60). Molecular assays for the rapid detection of isolates carrying specific resistance markers to tetracyclines (targeting mutations A965T, A967T) and spectinomycin (C1192A) were established (49, 50).

For the detection of decreased susceptibility to fluoroquinolones a PCR-RFLP and a TaqMan SNP real-time PCR assay were first available, both targeting the *parC* gene (revealing a nucleotide substitution G250A) (47, 48). MAMAs were also developed to target the mutations (C248T of *gyrA*, G239T and G250A of *parC*) detected in *M. bovis* field isolates with high fluoroquinolone MIC (50). In addition, several other mutations associated with elevated MIC to fluoroquinolone have been described in *gyrA* and *parC* (49, 60, 81, 82), and non-synonymous mutations in *gyrB*, *parE* and *topA* (type I DNA topoisomerase) have been linked to fluoroquinolone resistance using a GWAS approach (57). Besides the previously mentioned assays, alterations occurring in laboratory-derived antibiotic-resistant *M. bovis* mutants were also targeted with MAMAs and HRM tests (50). The identified mutations and published assays developed for the detection of the relevant point mutations are presented in Table 3 and Supplementary Tables 1–3.

For cattle *Mycoplasma* species other than *M. bovis*, antibiotic susceptibility data remain limited and insufficiently explored. Based on *in vitro* results, tilmicosin, erythromycin, oxytetracycline, and fluoroquinolones may be effective against *M. mycoides* subsp. *mycoides* (83, 84). However, the genetic background of antimicrobial susceptibility remains largely unexplored in this species with exception of *in vitro*-acquired streptomycin resistance in the vaccine strain T1, which has been attributed to a mutation in the *rplS* gene encoding the 30S ribosomal protein S12 (85). *M. bovirhinis*, a commensal microorganism of cattle that may exacerbate existing disease conditions caused by primary pathogens, has been found to carry the ParC substitution L80S in both field isolates and in laboratory-derived mutants with elevated MIC to fluoroquinolones (86).

Regarding small ruminant mycoplasmas, similar antimicrobial susceptibility profiles of *M. agalactiae*, *M. mycoides* subsp. *capri*, *M. capricolum* subsp. *capricolum* and *M. putrefaciens* were reported with fluoroquinolones and tetracyclines being the most effective agents against them (37, 87–92). Amino acid changes related to variations in fluoroquinolone susceptibility were found in GyrB, ParC and ParE of *M. agalactiae* (93), whilst an amino acid substitution in codon 83 of GyrA was described in *M. mycoides* subsp. *capri* (37, 89). Also, low susceptibility to macrolides and lincosamides had been linked to amino acid changes in L22 ribosomal protein, followed by the mutation of the 23S rRNA coding genes in *M. agalactiae* (94). Tetracycline resistance was not always associated with mutations in the 16S rRNAs, suggesting the existence of other resistance mechanisms in *M. agalactiae* yet to be deciphered (95). *M. ovipneumoniae* showed low MICs to tetracyclines, macrolides and lincosamides with only a few isolates requiring higher concentrations for inhibition. In contrast, moderately elevated MICs have been observed for florfenicol. Hot-spot mutations in the 16S rRNA could explain increased MIC for tetracycline, while besides a SNP in domain V of the 23S rRNA in one strain, no other genetic marker could be associated with the elevated MIC of macrolides, lincomycin or florfenicol (96). Despite the significance of Contagious Caprine Pleuropneumoniae, poor data on AMR profile or resistance-associated genetic markers of the etiological agent, *M. capricolum* subsp. *capripeumoniae*, are available (97, 98).

### 2.2 Swine mycoplasmas

The antimicrobial susceptibility profiles of the three main pathogenic swine mycoplasmas (*M. hyopneumoniae*, *M. hyorhinis*, and *M. hyosynoviae*) isolated from European countries are generally similar to each other. Most strains are currently susceptible to florfenicol, aminoglycosides, tetracyclines, fluoroquinolones, and pleuromutilins. However, decreased susceptibility to macrolides and lincosamides has been reported across all three species, with *M. hyorhinis* showing the most pronounced resistance (25, 99–104). Nevertheless, highly elevated MICs for these swine mycoplasma species against enrofloxacin, florfenicol, macrolides and lincomycin have been reported in Thailand emphasizing the importance of local surveillance programs in different geographical regions (105).

The mutations identified, along with the published assays designed to detect these specific point mutations, are listed in Table 3 and Supplementary Tables 1–3. In case of *M. hyopneumoniae* several resistance markers were identified for decreased susceptibility to macrolides, lincosamides and fluoroquinolones. *M. hyopneumoniae* has an intrinsic resistance to 14-membered macrolides, like erythromycin, which is explained by a point mutation G2057A of the genes encoding 23S rRNA (58). For other macrolides and lincosamides, mutations at nucleotide positions 2058 or 2059 in domain V of the 23S rRNA were described on several occasions (32, 58, 59, 99, 106, 107). A MAMA was developed to detect the transition at position 2059 (51). Decreased susceptibility to tiamulin was also associated with a transversion (A2058C) in domain V of the 23S rRNA (108). Resistance markers for fluoroquinolones were identified in the *parC* and *gyrA* genes. In ParC two amino acid changes at position 80 (from S to F or Y) and at position 84 (D to N) were identified, and MAMAs and a HRM test were designed to discriminate the corresponding mutations (51, 99, 109, 110). Recently, two other point mutations within the *parC* gene were linked to decreased susceptibility of *M. hyopneumoniae* to enrofloxacin (111). In GyrA, three amino acid changes were described (G81A, A83V and Q87G) and MAMAs were developed for their rapid detection (51, 99, 110).

For *M. hyorhinis*, markers associated with decreased susceptibility to macrolides, lincomycin and fluoroquinolones were identified. In the case of macrolides-lincosamides, a point mutation in domain V of the 23S rRNA was described. Transitions at position A2059G (112) or A2058G (53, 101) were identified in Korean and European isolates, respectively. In *in vitro* mutants, further mutations were described at domain II and V of the 23S rRNA (112). A MAMA was developed to detect the transition at position 2058 (53). For fluoroquinolones, based on a low number of field isolates (one or two), three mutations in ParC were associated with decreased susceptibility (S80Y, S81P, E84Q). Further mutations in the QRDR of *parC*, *parE* and *gyrA* genes were also identified in *in vitro* selected mutants (113).

In case of *M. hyosynoviae,* only one transition (G745A) in domain II of the 23S rRNA associated with decreased susceptibility to tylosin was identified in field isolates. Furthermore, three point mutations in domain V of the 23S rRNA were identified in *in vitro* mutants, located between the nucleotides 2058 and 2062 (59).

### 2.3 Avian mycoplasmas

Studies assessing the MIC values of antimicrobials for *M. gallisepticum* and *M. synoviae* field isolates have reported a global decline in susceptibility to multiple antimicrobial classes, including fluoroquinolones, tetracyclines, macrolides and lincosamides (26, 114–116). Table 3 and Supplementary Tables 1–3 provide an overview of the identified resistance-associated mutations and the published assays developed for their detection.

Several studies described mutations in and near the macrolide and lincosamide binding site in domains II and V of the 23S rRNA and in the L22 ribosomal protein-coding gene *rplV* both, in *M. gallisepticum* and *M. synoviae* (115, 117–124). Moreover, intrinsic resistance to 14-membered macrolides has been linked to a G2057A transition in the 23S rRNA coding gene in *M. synoviae* (121). Molecular tools, such as MAMAs for the detection of resistance markers in *M. synoviae* have been developed targeting the mutations in the L22 protein (Q90H) for macrolides and the A2059G transition in the *rrlA*/*rrlB* genes of 23S rRNA for macrolides and lincomycin (52). In *M. gallisepticum* mutants selected *in vitro* for elevated pleuromutilin MIC, several point mutations were identified within or near the binding site in domain V of the 23S rRNA (63). The genetic basis for decreased susceptibility to tetracyclines in poultry pathogenic mycoplasmas remains unclear. In *M. synoviae,* none of the mutations found in the 16S rRNA encoding genes were associated with elevated MIC, and the *tet*M gene was absent in the examined isolates (124). The fluoroquinolones target primarily the DNA gyrase genes in *M. gallisepticum* (125–128), while the topoisomerase IV genes are the main targets in *M. synoviae* (129, 130). Accordingly, point mutations and hot spot regions correlating with decreased fluoroquinolone susceptibility were described in the QRDR mainly of the ParC in *M. synoviae* and MAMAs were developed for the rapid detection of the mutations T80I/A (52, 116, 124, 130). Analysis of re-isolates of the live attenuated *M. synoviae* vaccine strain (Vaxsafe MS-H; Bioproperties Pty. Ltd.) revealed that the *parC* gene had a tendency to undergo spontaneous mutations resulting in amino acid substitutions at the previously described positions D84N, T85I, or D89N (124, 130, 131). Further mutations were also identified and served as targets for the molecular detection of resistance markers in *M. synoviae* in the ParE (S83F), GyrA (E133G) and GyrB (S401Y) proteins (52). Similarly, in *M. gallisepticum* isolates with elevated MIC for fluoroquinolones, resistance markers were identified in both GyrA and ParC proteins, while additional mutations in GyrB were associated with decreased susceptibility in this species (128, 132, 133).

A comprehensive MIC study was recently conducted on a diverse set of *M. iowae* isolates, which confirmed earlier findings about its low susceptibility to macrolides and revealed that the effect of lincomycin and spectinomycin is also limited on *M. iowae* (134, 135). Reports on the antimicrobial susceptibility profiles of the waterfowl pathogen *M. anserisalpingitidis* isolates, indicate elevated MIC values for most antibiotic classes used against this pathogen (136–138). The mutations G748A (domain II) and A2058G (domain V) in the 23S rRNA, and Q90H in the 50S ribosomal protein L22 were associated with elevated macrolide and lincomycin MICs in *M. anserisalpingitidis*, and MAMAs were designed for their rapid identification (54).

The growing concerns about AMR encouraged the examination of generally non-pathogenic but widespread avian mycoplasmas. These also exhibited a decreased susceptibility to macrolides, tetracyclines, and fluoroquinolones, similar to that observed in pathogenic species. Namely, decreased tylosin susceptibility was associated with a mutation in the L4 protein in *M. gallinarum* and in domain II of the 23S rRNA in *M. gallinaceum*. Although elevated MIC values for enrofloxacin and chlortetracycline were observed in these species, as well as in *M. pullorum* isolates, no additional genetic markers associated with antimicrobial susceptibility could be identified (123). The fact that similar mechanisms might influence the antimicrobial susceptibility of commensal mycoplasmas and the pathogenic ones—co-habiting the same organs in the same hosts—raises concern about possible interchanges of resistance-associated genetic elements among these species. Antimicrobial susceptibility studies on other avian pathogen mycoplasmas are rarely published and typically report MIC data for only a limited number of isolates; therefore, trends or general conclusions cannot be reliably drawn from them (17, 138, 139).

## 3 SWOT analysis of AMR molecular detection methods for livestock mycoplasmas

This analysis, conducted using a SWOT matrix presented in Table 4, provides insights into the potential and challenges of using molecular detection methods for the analysis of AMR in veterinary mycoplasmas. In veterinary practice, antimicrobials are crucial for minimizing economic losses due to *Mycoplasma* spp. infections. Enhancing AMR detection in mycoplasmas by use of molecular tests supports sustainable veterinary practices and aids in the broader effort to control AMR in both human and animal health, in line with the *One Health* approach, which highlights the interconnectedness of human, animal, and environmental health. The SWOT analysis underlines the important role that these methods can play in advancing veterinary medicine, improving animal health, and supporting public health initiatives according to the *One Health* approach.

**Table 4:** SWOT analysis of molecular tools for detection of AMR in veterinary mycoplasmas.

Strengths	Weaknesses
S1. Rapidity S2. High sensitivity and specificity S3. Direct clinical application S4. Multiplex testing options S5. Epidemiological insights S6. urveillance utility	W1. Target only known mutations W2. Miss unknown mechanisms W3. Limited phenotypic insight W4. Potential multiple mutations within resistance-determining regions W5. Regional clone variability W6. Detection on conserved sequences W7. Inconsistent assay sensitivity W8. Operational barriers W9. Cost of the techniques
Opportunities	Threats
O1. Protocol harmonization O2. Global data sharing O3. Ring trials O4. Wild-type vs. non-wild-type discrimination O5. Non-cultivable species testing O6. Database development O7. Bioinformatics integration O8. AI-assisted AMR detection	T1. Evolving resistance mechanisms T2. Non-validated methods T3. Inconsistent mutation numbering T4. Lab mutants vs. field strains T5. Genomic complexity barriers T6. High mutation rate and HGT T7. Constant tool updates are needed

HGT, Horizontal Gene Transfer; AI, Artificial Intelligence.

Strengths and Weaknesses are typically considered as internal factors inherently under the control of a system, while Opportunities and Threats are external factors that need to be examined to define future perspectives for AMR surveillance (92).

### 3.1 Strengths

Cultivating mycoplasmas *in vitro* is challenging, requiring specialized laboratories due to specific growth conditions and slow replication. Additionally, the absence of standardized procedures for traditional culture-based methods (such as variability in culture media, result presentation, and lack of quality control strains) makes comparing studies on mycoplasma AMR complicated (18). In contrast, PCR-based methods for determining AMR provide significant advantages in speed (Table 4, bullet point S1) and reliability, as they directly detect the presence or absence of resistance-associated mutations, thereby facilitating easier interpretation. The reduced detection time compared to traditional culture-based methods helps in the choice of adequate antimicrobials and, consequently, increases therapeutic efficiency (140). Moreover, PCR-based tests enable highly sensitive and specific detection of resistance-associated mutations, even at low abundance (Table 4, bullet point S2) (47–54). Therefore, making them suitable for direct application to clinical samples (Table 4, bullet point S3). Many molecular platforms support the simultaneous detection of multiple resistance genes or pathogens in a single test, thereby saving time and resources (50–52) (Table 4, bullet point S4).

Moreover, molecular methods represent convenient tools for the surveillance of genetic mutations associated with AMR, as they can be used as an indirect method providing information of strain relatedness and revealing epidemiological investigations to control possible outbreaks involving resistant bacteria (141) (Table 4, bullet point S5). For instance, recent studies have connected reduced antimicrobial susceptibility to the dissemination of a certain multi-resistant genotype of *M. bovis* (142) as well as a specific genetic clonal complex of *M. gallisepticum* (115).

In addition, molecular characterization can identify the specific genetic mechanisms underlying resistance, such as particular mutations (e.g., G748A in domain II of the 23S rRNA, which causes macrolide resistance) and SNPs (e.g., A2058G or A2059G in domain V) associated with elevated MIC values for macrolides and cross-resistance to lincomycin in *M. bovis* (49, 55, 60). This knowledge may guide a tailored treatment strategy (Table 4, bullet point S3) and supports the monitoring of multi-AMR over time (Table 4, bullet point S6).

### 3.2 Weaknesses

Although the use of genetic methods in the determination of antimicrobial susceptibility could provide valuable guidance for antimicrobial therapy, the presence of factors that can complicate the genetic detection of certain mechanisms (i.e., efflux pump) should be considered as well (30, 37). The reliability of PCR-based molecular techniques depends on known genetic determinants of resistance and may miss novel mutations (Table 4, bullet point W1) or unknown resistance mechanisms (Table 4, bullet point W2). Moreover, PCR-based methods may not fully indicate phenotypic resistance, which can be influenced by levels of gene expression, regulatory mechanisms, or post-transcriptional modifications (141) (Table 4, bullet point W3).

Identifying mutations responsible for AMR is also challenging, as they often occur alongside other mutations within the resistance determining region (Table 4, bullet point W4), making it difficult to evaluate their individual effects (Supplementary Tables 1–3). This poses a particular challenge for mycoplasmas, which have been described as “constitutive mutators” due to their limited DNA repair capacity (143), and currently exhibit one of the highest nucleotide substitution rates (144). As a result, tests targeting specific mutations can fail due to nearby spontaneous mutations. Additionally, possible differences in selection pressures acting on natural clones of geographically distinct origin driven by differing antimicrobial usage, may lead to regional heterogeneity in the distribution of AMR-associated mutations. Nevertheless, current genomic data remain insufficient to fully substantiate these differences. Accordingly, while PCR-based tools can remain globally standardized, their validation and interpretation should be contextualized to regional antimicrobial usage patterns and the prevailing clonal populations (Table 4, bullet point W5).

PCR-based methods face a biotechnological challenge in detecting AMR-related point mutations within highly conserved regions of the mycoplasma genome, such as 16S rRNA and 23S rRNA, which often exist in multiple copies. Cross-reactivity with other mycoplasma species has been frequently reported in MAMA and HRM assays targeting these regions (Supplementary Tables 4A,B) (50–52) (Table 4, bullet point W6). The sensitivity of these assays varies, typically ranging from 10^2^ to 10^4^ copies/μL, allowing the direct analysis of clinical samples. However, certain assays (HRM for *M. hyopneumoniae* (51)) require higher bacterial load (10^5^–10^6^ copies/μL), to yield reliable results, limiting their applicability in low-titer samples. In such cases, isolating the pathogen may be necessary before testing (Supplementary Tables 4A,B) (Table 4, bullet point W7).

Operational factors like total turnaround time (including sample transport, processing, and reporting), requirement for specialized equipment/training, and ease of integration into routine veterinary diagnostic laboratory workflows and the expected number of analyses significantly affect the practical adoption of molecular methods, especially in resource-limited settings (Table 4, bullet point W8). Besides, molecular detection technologies are generally more costly than phenotypic methods, particularly in low-resource settings due to the cost of specialized equipment, reagents, and technical expertise (Table 4, bullet point W9), which is often balanced by the significant time saved in obtaining results (46).

### 3.3 Opportunities

As there is only a limited number of studies comparing the phenotypic and genotypic MIC profiles of veterinary pathogen mycoplasmas, future opportunities for test development in molecular detection of AMR lay in creating and harmonizing protocols for different methods (Table 4, bullet point O1) and evaluating them using a wider variety of strains (46). MyMIC, a network for standardization of diagnostics, AST and clinical interpretation for animal mycoplasmas, includes 22 laboratories from 18 countries and possesses the antibiotic susceptibility profiles—often with the whole genome sequences—of hundreds of mycoplasma strains from their collections. Analyzes of such databanks could allow the identification of mutations associated with a decrease in antimicrobial susceptibility. By facilitating a large-scale, standardized, and coordinated study, MyMIC has the potential to provide comprehensive insights into the diversity of AMR mutations and underlying resistance mechanisms. Moreover, molecular characterization of AMR determinants through global collaborations and partnerships increases the accessibility and affordability of genotypic resistance testing (46) (Table 4, bullet point O2). Nevertheless, international harmonization of testing protocols, interpretive criteria, and quality control measures, supported by inter-laboratory ring trials, is crucial to ensure the reliability, comparability, and global utility of molecular AMR data for livestock mycoplasmas (145) (Table 4, bullet point O3). To date, the uptake of molecular techniques developed by one has been limited among other research teams.

Beyond established PCR-based methods, isothermal amplification techniques like Loop-Mediated Isothermal Amplification (LAMP) offer potential for rapid, field-deployable detection of resistance markers (146), although validation for mycoplasma AMR is currently limited. Droplet digital PCR has also emerged as a promising approach for precise and sensitive detection of AMR determinants (147). Next-generation sequencing (NGS), particularly targeted amplicon sequencing or rapid Nanopore sequencing, enables comprehensive detection of known mutations and discovery of novel markers within a single assay.

Identification of mutations associated with a decrease in antimicrobial susceptibility enable the discrimination of wild-type and non-wild-type populations (Table 4, bullet point O4) and so can also be a tool to help setting interpretation criteria for AMR for mycoplasma species (148). Molecular tools can refine the interpretation of MIC distributions by identifying variations within seemingly monomodal populations. The wild-type population represents isolates without phenotypically detectable acquired resistance. Although no strict threshold defines the shift from wild-type to non-wild-type, the ECOFF is used to mark the highest MIC in the wild-type population. Molecular methods help distinguish non-wild type population by detecting subtle resistance-related variations that are not evident in traditional phenotypic tests (148).

In the special case of non-cultivable hemotropic mycoplasmas (Table 1) antimicrobial treatments, -particularly with tetracyclines-, is commonly used (149). However, several studies have evidenced treatment failures [e.g., *M. wenyonii* and *M. ovis* (150–152)]. Since traditional *in vitro* AST is not feasible for non-cultivable species, molecular techniques have become crucial for studying the antimicrobial susceptibility of these organisms (Table 4, bullet point O5).

The development of comprehensive databases of *Mycoplasma* species, which contain the relevant sequence targets, along with the application of suitable bioinformatic tools to extract pertinent information from sequence data, is essential for accurate interpretation (Table 4, bullet point O6). Currently, several AMR databases or bioinformatics pipelines exist, developed for other bacteria (such as the Comprehensive Antibiotic Resistance Database (CARD), ResFinder, and Pathogenwatch), which may serve as a source of inspiration for customizing a mycoplasma-database. Single web solutions should be iterative and enhanced by regular, validated updates of newly identified gene sequences and point mutations. Advances in sequencing technologies and bioinformatics could enhance the scope and cost-effectiveness of molecular methods (Table 4, bullet point O7), potentially leading to more comprehensive and affordable AMR detection (141).

Recent advances in artificial intelligence (AI) and machine learning offer promising opportunities for rapid detection and prediction of AMR in bacterial pathogens, including *Mycoplasma* species. AI can leverage genomic or phenotypic data, such as whole genome sequence (WGS), microscopy, or MALDI-TOF MS, to identify resistance-associated mutations and classify isolates as susceptible or resistant within hours (153). Although not yet widely applied to mycoplasma, these approaches hold potential for improving antimicrobial stewardship and supporting the *One Health* framework. A key limitation is the need for large, well-annotated datasets; for mycoplasmas, the limited availability of sequences and phenotypic data currently restricts broader implementation, emphasizing the importance of expanding high-quality datasets in future studies (Table 4, bullet point O8). Future integration of rapid point-of-care molecular diagnostics with machine learning algorithms for real-time AMR prediction based on genomic markers holds transformative potential for on-farm decision support.

### 3.4 Threats

The continuous evolution of resistance mechanisms, including mutations that are not yet detectable by current molecular methods, could promote AMR spread and hinder detection (Table 4, bullet point T1). Use of non-validated assays risks misdiagnosis, leading to inappropriate antimicrobial use (treatment failure or unnecessary broad-spectrum use), exacerbating AMR, and generating misleading surveillance data (Table 4, bullet point T2). Without regular validation that accounts for the rapid genomic evolution of mycoplasmas, these methods may produce inconsistent or unreliable results. Ensuring rigorous validation before commercial release is therefore essential to maintain accurate detection, guide appropriate treatment decisions, and support reliable surveillance (154). Key validation parameters include analytical sensitivity (limit of detection for mutant alleles in mixed populations), analytical specificity (ability to distinguish target mutations from closely related sequences/non-targets), diagnostic sensitivity/specificity (performance compared to phenotypic AST), and reproducibility (intra- and inter-laboratory consistency). Regular re-evaluation of these parameters is critical to maintain assay robustness and diagnostic accuracy over time.

A comprehensive quality check is necessary for the sequences that are already available to ensure their accuracy and reliability. Quality control process starts with factors such as the choice of the strains, culturing protocols and laboratory environment, followed by subsequent steps that include assessing coverage depths and identifying potential errors, such as base calling inaccuracies, contamination, or incomplete data. Performing this quality control is essential to assess whether the sequence data have reached a suitable standard, as resistance genes or mutations might be missed in sequences of poor quality (148). Besides, the low genomic complexity of mycoplasmas, characterized by their high AT content, complicates sequence-based analysis (13).

In most studies, the *E. coli* numbering system is commonly employed to annotate mutations in *Mycoplasma* species. However, using *E. coli* strain K12 substrain MG1655 as a reference for describing mutations in other bacteria species can lead to misunderstandings due to the lack of a standardized approach for analysis (Table 4, bullet point T3). The length, nucleotide sequence, and copy number of the 23S rRNA gene exhibit considerable variation among bacterial species (155). Moreover, even within the *E. coli* K12 MG1655 genome, differences are observed among the seven copies of the 23S rRNA gene, with *rrlA* measuring 2,905 base pairs and the other six copies measuring 2,904 bp each leading to different numbering. Due to this one base pair length difference between the copies of the 23S rRNA gene in *E. coli*, positions such as 2,058 and 2,059 related to decreased susceptibility to macrolides and lincomycin in *M. bovis,* may be inconsistently identified in previous publications (49, 55, 56, 60, 66, 76–80). A significant challenge in comparing mutations across studies is the inconsistent use of numbering systems (e.g., *E. coli* versus native mycoplasma numbering). Besides, alternative numbering systems (according to reference mycoplasma genome) have been introduced recently, further complicating cross-species comparisons (52, 57). To address this, we recommend reporting mutations both with respect to the widely used *E. coli* numbering system, which facilitates comparisons across bacterial species, and the native gene sequence of the type strain for each *Mycoplasma* species. To improve clarity and reproducibility in mutation reporting, we recommend mapping *E. coli* numbering to the native *M. bovis* 23S rRNA coordinates by pairwise (or multiple) sequence alignment of the full 23S rRNA sequences and converting coordinates by counting aligned, non-gap positions. As an example, the mutation associated with macrolide resistance in *M. bovis* can be mapped by: (1) retrieving the full-length 23S rRNA sequence of *E. coli* K-12 MG1655 (specifying the *rrl* copy used: GenBank Accession number: U00096.3, *rrlB*) and that of the *M. bovis* PG45 type strain (GenBank Accession number: CP002188.1); (2) perform a global alignment (e.g., MAFFT); (3) walking through the aligned columns while incrementing positional counters only for non-gap positions; (4) we recommend reporting mutations in the following dual format: A2058G [*E. coli*]/A2057G [*M. bovis* PG45]. This ensures transparency and allows direct comparison across studies using different reference frameworks. Although we were unable to present both numbering systems consistently throughout this review due to limited or conflicting published data, providing the type strain reference sequence is strongly advised, as it minimizes confusion, enables accurate aggregation of data across studies, and ensures clarity when interpreting resistance-associated mutations. Furthermore, making the full gene sequence data publicly available, in addition to the annotated point mutations, is important as it contributes to the development of new diagnostic methods and predictive models.

The assays targeting mutations detected in laboratory-derived mutants possess minor importance in routine diagnostics (Table 4, bullet point T4). *In vitro* mutants are selected under controlled conditions with sub-inhibitory antibiotic concentrations, which do not represent the natural selection pressures on field clones (60, 63, 125). As a result, mutations observed in laboratory mutants may not accurately predict resistance in wild-type strains. Therefore, molecular tools targeting resistance markers described in natural clones offer more reliable results for diagnostics, although their applicability may be influenced by distinct selection pressures due to differences in antimicrobial treatments by geographical regions.

The high frequency of nucleotide substitutions –even in the absence of selective pressure– and the presence of mycoplasma chromosomal transfer in mycoplasmas present unique challenges for AMR monitoring and diagnostic efforts (156) (Table 4, bullet point T5). Mycoplasmas, with their small genomes and fast-evolving nature, could rapidly acquire mutations that can confer resistance to antimicrobials (Table 4, bullet point T6). Additionally, conjugative exchanges of large chromosomal fragments that equally affected all parts of the chromosome via an unconventional mechanism have been described, suggesting that the whole mycoplasma genome, including genes with mutations conferring resistance, is potentially mobile (43). Due to this dynamic genetic landscape, molecular tools used for detecting resistance in mycoplasmas must be regularly updated and adapted to account for emerging resistance mechanisms (Table 4, bullet point T7). Continuous refinement of these tools is essential to ensure accurate detection and effective surveillance of AMR in mycoplasmas, as recommended by studies on microbial evolution and resistance monitoring.

## 4 Focus on genome wide association studies (GWAS)

**Figure 2:**
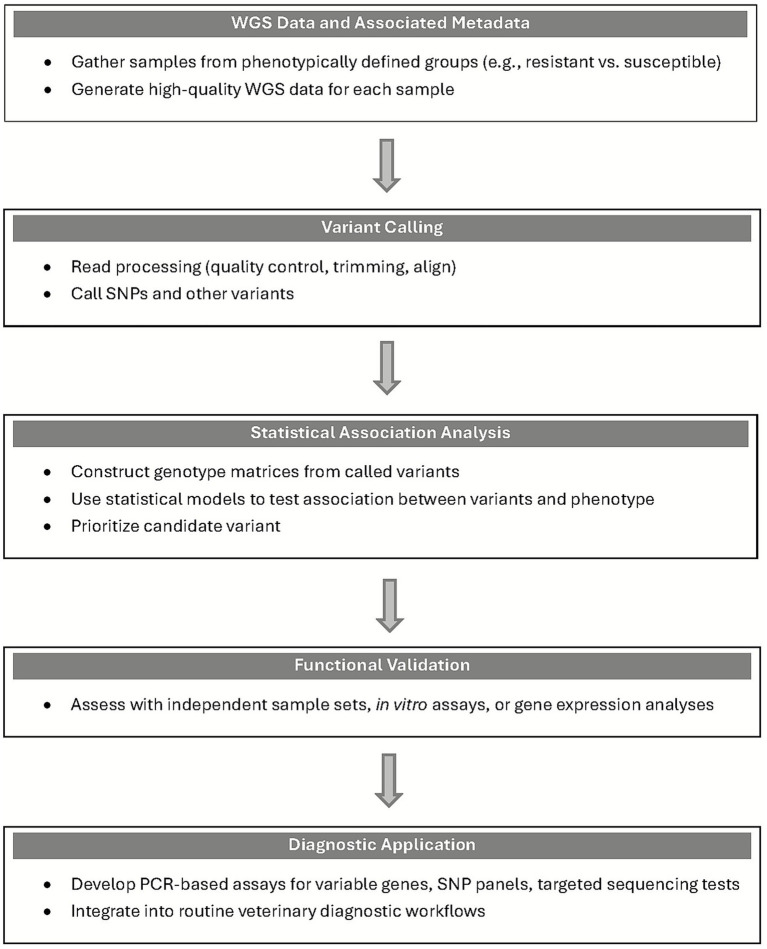
Schematic representation of GWAS workflow and its role in informing diagnostic development. WGS, whole genome sequence, PCR, polymerase chain reaction; SNP, single nucleotide polymorphism.

GWAS identify correlations between genetic variants and phenotypes using complete or draft genomes (Figure 2). Phenotypic traits can be binary, categorical, or continuous. The tested markers can also take many forms, such as clusters of ortholog genes (COGs), SNPs, k-mers (nucleotide sequence with “k” length) and genes. SNPs represent single nucleotide changes found in at least 1% of genomes and are useful for identifying causal variants, though complex traits often require broader context. K-mers provide higher resolution but are harder to interpret. Due to the multiple statistical testing, the analysis includes statistical corrections and stringent *p*-value thresholds (typically ≤10E-8). Various tools are currently available for performing GWAS analysis in bacteria, including seer, pyseer, DBGWAS, GWAMMAR, Scoary, bugwas, and Phenotype Seeker. These software packages use different statistical methods like fixed effect generalized linear regression, odds ratio, hypergeometric test, Fisher’s exact test, binomial test, linear mixed model and many others. These statistical tests compare the variation found in the genomes of the samples with the phenotypes of the samples (157, 158).

High-quality genomes are essential, as low-quality data can introduce bias into the results. The quality of sequencing and whole genome data can vary significantly between platforms, making accurate comparisons challenging. Common high-throughput short-read sequencing platforms are Illumina and Ion Torrent machines, which produce large amounts of sequence data compared to the traditional Sanger sequencing technology. Read length in most cases are shorter (100–400 bp) than the genes conferring resistance to antimicrobial agents. Nanopore sequencing platforms enable real-time long-read sequencing and have traditionally exhibited a higher error rate compared to the Illumina platform (55, 140, 141). Recently, for certain bacterial species, Oxford Nanopore Technologies (ONT) can generate near-complete microbial genomes from isolates or metagenomes without the need for short-read or reference polishing (159). Additionally, the use of taxon-specific base calling has been shown to improve consensus accuracy and genome completeness for *M. bovis* field strains (160).

A major advantage of the GWAS approach is its ability to identify novel genetic markers beyond previously known or targeted regions, including those outside coding sequences. Even if the relationship between the genetic element associated with the phenotype is not based on cause and effect, the association could be a target for new diagnostic methods (157). WGS analysis also offers the possibility to perform fast *in silico* reanalysis on already sequenced isolates. It should be noted that WGS analysis methods, similar to PCR-based methods, may not accurately reflect phenotypic resistance, which can be influenced by gene expression levels, regulatory mechanisms, or post-transcriptional modifications (141).

The relatively few studies conducted on mycoplasmas may be due to the need for a large number of strains (usually more than a hundred and up to thousands), the high cost and time-consuming WGS analyses of the samples, and a more specific set of skills. Although sequencing costs continue to decline, WGS of *Mycoplasma* strains remains considerably expensive (typically ranging from approximately $72 to $470 per strain) depending on sequencing depth and platform used (161). This represents a substantial expense for large-scale population studies of *Mycoplasma*. Furthermore, a limitation of GWAS is the risk of false associations, often resulting from insufficient statistical power, the complexity of polygenic inheritance, and variable penetrance of genetic variants (56).

So far, among animal mycoplasmas, GWAS has been conducted in the context of antimicrobial susceptibility only for *M. bovis* (55–57). *M. bovis* studies identified non-synonymous mutations in the 50S and 30S ribosomal proteins linking to macrolide resistance. Phenicols and pleuromutilins share similar antimicrobial mechanisms with macrolides, making the observed similarities in studies unsurprising. Florfenicol GWAS identified mutations in 23S rRNA (A2059G, A2060C) and in 50S ribosomal protein L4 of *M. bovis* clinical isolates (56). Tetracycline GWAS identified SNPs in the proteins of the rRNA subunits (56, 57), while gentamicin GWAS described two potentially resistance-associated substitutions (A1408G and G1488A) of 16S rRNA genes (Supplementary Tables 1–4) (55).

In addition to the well-documented genetic alterations associated with resistant phenotypes, GWAS conducted on *M. bovis* for all antimicrobial classes have revealed novel nucleotide variants in non-target genes, including those encoding ABC transporters, tRNA-ligases, peptidases, and transposases. These findings suggest that several mutations could result in one AMR phenotype (55–57). Functional validation is required to confirm the roles of these proteins in resistance mechanisms. In addition, further studies are needed to assess GWAS in other *Mycoplasma* species. In summary, while GWAS offers powerful discovery potential, its application to mycoplasma AMR faces challenges: (1) requirement for large, well-phenotyped strain collections which are often scarce; (2) high costs and bioinformatic expertise needed for robust whole-genome sequencing and analysis; (3) the polygenic nature of some resistance phenotypes and the confounding effects of population structure requiring sophisticated statistical models and careful interpretation; (4) Validation of statistically associated markers with functional studies, which are particularly complex in slow-growing, fastidious mycoplasmas.

More recently, Bokma et al. (55) demonstrated the potential of quick high-quality Nanopore sequencing combined with GWAS analysis for assessing phenotypic ECOFF thresholds and the rapid identification of *M. bovis* strains with acquired resistance. Beyond refining ECOFF determination, this integrated approach highlights how GWAS data can be translated into practical diagnostic applications. The resistance-associated variants identified through such analyses provide a valuable basis for developing targeted PCR-based detection assays, facilitating the transition from large-scale genomic discovery to routine molecular diagnostics.

## 5 Perspectives

Molecular detection tools offer interesting solutions for the rapid and accurate identification of AMR in livestock mycoplasmas. Current PCR-based methods enable the detection of known resistance associated alterations but remain limited to pre-characterized mutations and could be failed by the apparition of nearby, not targeted mutations. The growing adoption of WGS approaches is transforming AMR research in mycoplasmas. As these technologies improve and become more accessible, they are increasingly enabling accurate and rapid detection of resistance determinants, broadening the scope beyond point mutations in specific genes, thus providing a more comprehensive view, uncovering novel resistance mechanisms and facilitating genome-wide surveillance across diverse populations. Finally, NGS sequencing is facilitating the development of rapid and sensitive molecular diagnostics for AMR detection, allowing for more targeted therapy and improved antimicrobial stewardship in veterinary medicine.

To fully realize the potential of GWAS for AMR prediction in mycoplasmas, several key advancements are necessary. First, standardizing mutation reporting should include both *E. coli* numbering and native mycoplasma numbering, and full gene sequences must be made publicly accessible to support data aggregation, diagnostic development, and predictive AMR modeling. Then, the developed molecular AMR assays require rigorous validation, encompassing analytical sensitivity and specificity, diagnostic sensitivity and specificity, and reproducibility both within and between laboratories to ensure reliability over time. Also, taking into consideration that at present no clear geographic association has been identified between the occurrence of specific mutations and the region of origin (likely due to the limited number of available sequence data), the potential for significant geographic variation in resistance patterns driven by differing antimicrobial usage makes the establishment of robust local and national AMR surveillance programs for livestock mycoplasmas essential for effective stewardship and targeted therapy. Expanding collections of geographically diverse isolates and improving access to bioinformatics expertise would further strengthen these efforts, enabling the identification of region-specific resistance markers that could serve as the basis for tailored molecular detection assays. Translating such findings into targeted PCR-based diagnostic tools would facilitate rapid, cost-effective surveillance of resistance patterns, while integrating genomic discovery with applied diagnostics would enhance both global and regional management of AMR in mycoplasmas. To improve applicability in resource-limited settings, future advancements should focus on cost-reduction strategies. Promising directions include miniaturized PCR devices, and multiplex assays capable of detecting multiple resistance markers simultaneously (162). Moreover, open-access databases and collaborative networks can lower analysis costs and improve data sharing, supporting broader integration of molecular tools into field diagnostics and enabling real-time AMR monitoring in farm environments on known mutations.

Additionally, machine learning algorithms integrated with bioinformatics could enhance the interpretation of complex resistance data (163). However, translating genomic findings into practical clinical guidelines remains a challenge, requiring coordinated efforts to support antimicrobial stewardship and AMR control strategies in veterinary settings. A key component of this process is the interpretation of NGS data, which relies on curated databases such as the CARD, ResFinder, and Pathogenwatch. While these resources provide extensive repositories of resistance genes and mutations, mycoplasma-specific curation is still limited, highlighting the need for ongoing efforts to ensure accurate and clinically actionable insights. In this context, developing or expanding a dedicated mycoplasma-specific database would facilitate the integration of newly identified AMR-associated mutations. As molecular tools continue to evolve, they will play an increasingly critical role in guiding targeted therapies and limiting the spread of resistance in mycoplasma infections.

To fill in all these perspectives, the MyMIC network represents a significant asset, bringing together valuable biological resources—including hundreds of isolates with corresponding MIC data—and experts in mycoplasma AMR. Currently, MyMIC is also contributing to the standardization of phenotypic AST methods and the definition of ECOFF, which serve as the foundation for the ongoing validation of molecular techniques.

## 6 Conclusion

Enhancing the detection of AMR in livestock mycoplasmas is crucial for promoting responsible antimicrobial use, especially when combined with timely and precise training for professionals. By improving detection methods, we can better and rapidly identify resistant strains, which helps veterinarians make informed decisions about treatment options. The aim of molecular AMR detection is to identify existing resistance traits, whereas prediction involves forecasting the emergence of resistance or discovering novel mechanisms. While PCR-based detection methods provide insight into current resistance patterns, GWAS are better suited for the prediction of future resistance markers, as they analyze genetic variations across the entire genome, linking these to resistance phenotypes and uncovering previously unknown determinants of resistance. This approach not only optimizes the effectiveness of antimicrobials in animal health but also reduces the selective pressure that contributes to the development of resistance in other bacteria.

## Footnotes

1 Jaÿ, M, Klose, SM, Bottinelli, M, Becker, CAM, Bokma, J, Boyen, F, et al. Advancing standardization of diagnostics and antimicrobial susceptibility testing for pathogenic mycoplasmas of livestock origin: insights from the MyMIC network. *BMC Vet Res*. doi: 10.1186/s12917-025-05154-4 (accepted).
